# Perceived health risks associated with the use of tobacco and nicotine products during the COVID-19 pandemic

**DOI:** 10.18332/tid/136040

**Published:** 2021-06-09

**Authors:** Yong Yang, Eric N. Lindblom, Ramzi G. Salloum, Kenneth D. Ward

**Affiliations:** 1 School of Public Health, The University of Memphis, Memphis, United States; 2 O’Neill Institute for National and Global Health Law, Georgetown University Law Center, Washington, United States; 3 Department of Health Outcomes and Policy, University of Florida, Gainesville, United States

**Keywords:** perceived risk, COVID-19, tobacco/nicotine products

## Abstract

**INTRODUCTION:**

The perceived health risks of tobacco products may change during the ongoing COVID-19 pandemic. This study aimed to examine the perceived risks of tobacco use on COVID-19 infection and severity, and possible COVID-related changes in perceptions of tobacco use and overall health.

**METHODS:**

We conducted an online survey of adults in the United States in June 2020 (n=2097). The survey covered cigarettes, cigars, e-cigarettes, and hookah. We also assessed changes in the use of any of the four tobacco products. Multivariate logistic regression models were used to estimate the odds of agreeing with the perceived risks for each risk and each product, with the adjustment of demographic and COVID-19 related variables.

**RESULTS:**

For all four tobacco products, the perceived risks to general health were slightly higher during the pandemic than before the pandemic (77% vs 79.5% for cigarettes) and the perceived risk of COVID-19 severity was larger than the perceived risk of COVID-19 infection (73.3% vs 56.2% for cigarettes). All risk measures varied with tobacco products consistently, with the risks highest for cigarettes, then cigars, followed by e-cigarettes and hookah. Females and people with higher income or education were more likely to endorse the risks of tobacco use than their counterparts. People who perceived higher risks of using cigarettes (OR=1.65; 95% CI: 1.20–2.27) and cigars (OR=1.63; 95% CI: 1.17–2.27) to COVID-19 severity were more likely to have decreased or quit their use.

**CONCLUSIONS:**

Tobacco/nicotine use was perceived to increase the risk of COVID-19 severity and the perceived risk of tobacco/nicotine use to general health was high during the pandemic, particularly for cigarettes. The change of perceived risks appeared to be prompting harm-reducing changes in tobacco product use.

## INTRODUCTION

Perceptions of health risks and harms are associated with tobacco product experimentation, initiation, frequency, and intensity of use, as well as quit attempts^1-3^. In tobacco regulatory science, findings of health risk misperceptions that can promote harmful tobacco use behaviors are used to prompt and inform corrective action through new public education efforts or new requirements relating to product characteristics (e.g. flavors or appearance), product packaging, and labeling design, advertising and other marketing, warning labels, and educational package inserts^4,5^.

Perceived health risks of tobacco products may change during the ongoing COVID-19 pandemic, given the uncertainty surrounding the association between smoking, vaping, and COVID-19 incidence and outcomes. Preliminary evidence indicates that although smoking may be associated with a higher mortality rate and suffering more severe consequences of COVID-19^6,7^, it is not clear if current smoking is associated with a reduced risk of COVID-19 infection^8-10^. This initial research seems to contradict the hypothesis that smokers are at higher risk of disease severity due to mechanisms such as pre-existing lung disease or reduced lung capacity or higher rates of various other diseases among smokers compared to non-smokers, and have an increased transmission risk due to greater contact between fingers and lips and inhaling deeply near one’s fingers. These seemingly controversial findings, together with misleading information on social media, may confuse the public, particularly smokers. Similarly, the influence of other tobacco product use such as cigars, e-cigarettes, and hookah on COVID-19 is largely unknown. The uncertainty and controversy may impact the perceived risk of tobacco use among both tobacco users and non-users.

Research on the perceived health risks of tobacco/ nicotine use during COVID-19 pandemic is currently limited. Initial evidence showed the majority of people who used cigarettes and e-cigarettes believed that their tobacco/nicotine use increased their risk of COVID-19 in general^11,12^. Another study found that the majority of people who used tobacco/nicotine believed that their tobacco/nicotine use did not increase the risk of COVID-19 infection but increased the severity of COVID-19 if infected^13^. Further, among tobacco/nicotine users, a high perception of the risk of tobacco/nicotine use to COVID-19 was associated with a reduction in tobacco/nicotine use or an increase in the desire to quit tobacco/nicotine use^11,14^. However, these studies were conducted either in relatively small regions or having a small sample size thus limiting the generality of their findings.

This study aimed to examine the perceived risks of tobacco/nicotine use on COVID-19 infection and COVID-19 severity, as well as any possible COVID-related changes in perceptions of tobacco-nicotine use and overall health harms and risks. Specifically, we examined how perceived risks were distributed within the population and varied by tobacco products, related factors, and their influence on tobacco use behaviors.

## METHODS

We conducted a survey of adults in the United States using Amazon Mechanical Turk (MTurk, https:// www.mturk.com/), an online crowdsourcing platform to conduct surveys. Inclusion criteria were: 1) ever use (as of 31 December 2019) of at least one tobacco product among the four categories of cigarettes, cigars (including cigarillos and small cigars), e-cigarettes, and waterpipe (i.e. hookah); 2) residence in the US; and 3) 90% or above approval rating from previous MTurk tasks. Eligible participants were given access to the survey, hosted by Qualtrics (https://www. qualtrics.com/). The survey was active in MTurk between 8 and 18 June 2020. Each eligible participant was compensated $0.25. The Institutional Review Board at the University of Memphis approved this study.

In addition to demographic information (age, gender, race/ethnicity, educational level, and household income), participants reported the COVID-19 status of themselves and their family, whether they had experienced a COVID-19 death among family members, friends, or colleagues, and the zip-code where they lived. Using the home zip-code, the urbanization level of the neighborhood was classified as urban, suburban, or rural, using the rural-urban commuting area (RUCA) codes^15^.

The survey focused on four types of tobacco-nicotine use: e-cigarettes or smoking either cigarettes, cigars, or hookah – and asked about the perceived risks of each on: 1) likelihood of COVID-19 infection; 2) severity of COVID-19 if infected; and 3) general health. For COVID-19 infection, participants were asked: ‘Do you agree that the use of each of the following tobacco products will increase the risk of getting COVID-19?’, and the response options were based on a 5-point Likert scale (strongly agree, agree, neither agree nor disagree, disagree, and strongly disagree). For COVID-19 severity, the question was: ‘If a person gets COVID-19, do you agree that the use of each of the following tobacco products will make him/her sicker, or sicker for a longer period of time?’. For general health, we assessed perceived risk both before and during the COVID-19 pandemic. The questions were: ‘Before the COVID-19 pandemic, for example, during the year of 2019 in general, to what extent did you agree that the use of each of the following tobacco products causes serious illness is very convincing?’ and ‘Right now, to what extent do you agree that the use of one tobacco product causes serious illness is very convincing?’.

We also assessed changes in the use of any of the four tobacco products during the COVID-19 pandemic, including new initiation, cessation, switching, or changes in consumption levels.

To analyze the data, we first summarized the percent of participants who agreed or strongly agreed with the perceived risks for each tobacco/ nicotine product. Second, multivariate logistic regression models were used to estimate the odds of agreeing with the perceived risks for each risk and each product. Finally, multivariate logistic regression models were used to estimate how the perceived risks influenced the odds of decreasing or quitting the use of each tobacco product compared with increasing or initializing it. All multivariate logistic regression models were adjusted by demographic variables including gender, age, race/ethnicity, educational level, and household income, urbanization level of the neighborhood, COVID-19 related variables such as if any COVID-19 infection for self and family and if any COVID-19 death among social network, and the use status of the specific tobacco/nicotine product in 2019. Analyses were performed using SAS 9.4 (SAS Institute, Inc., Cary, NC).

## RESULTS

Among the 2097 participants who completed the survey, 53% were male, 57% were aged 18–34 years, 63% were White, 10% were Black, 16% were Hispanic, 8% were Asian, 60% had at least a Bachelor’s degree, and 81% lived in urban areas. Most (91%) participants and their family members did not have any COVID-19 symptoms, and 22% reported a COVID-19 death among family members, friends, or colleagues. Among the participants, the prevalence of ever using (considering both exclusive use and co-use or poly-use with other tobacco products) cigarettes, cigars, e-cigarettes, and hookah in 2019 was 67%, 23%, 39%, and 16%, respectively (Supplementary file Table S1).

As Table 1 shows, for all four products, the perceived risk of increased severity of COVID-19 if infected due to tobacco use (i.e. percent of participants who agreed or strongly agreed) was larger than the perceived risk of COVID-19 infection: the range of the former was 45.7–56.2% while the range of the latter was 62.8– 73.3%. It should be noted that the average value of perceived risks of COVID-19 infection were roughly 50%, with perceived risks associated with cigarettes and cigars above 50% and risks of e-cigarettes and hookah below 50%. Second, for all tobacco products, the perceived risks to general health during the pandemic were slightly higher than the perceived risk to general health before the pandemic (79.5% vs 77.0% for cigarettes). Third, all risk measures varied with tobacco products consistently. The risks were highest for cigarettes, then cigars, followed by e-cigarettes and hookah.

**Table 1:** Participants who agreed or strongly agreed with the risks of using major tobacco/nicotine products (N=2097)

*Tobacco/nicotine products*	*Perceived risk of using the tobacco/nicotine product, n (%)*			
	*To COVID-19 infection*	*To the severity of COVID-19 if infected*	*To general health*	
			*Before the pandemic*	*During the pandemic*
Cigarettes	1179 (56.2)	1537 (73.3)	1615 (77)	1667 (79.5)
Cigars	1076 (51.3)	1447 (69)	1474 (70.3)	1516 (72.3)
E-cigarettes	958 (45.7)	1317 (62.8)	1277 (60.9)	1311 (62.5)
Hookah	960 (45.8)	1327 (63.3)	1164 (55.5)	1225 (58.4)

As Tables 2, 3 and 4 show, females were more likely than males to perceive tobacco use as a risk factor for COVID-19 infection (cigarettes: OR=1.2; 95% CI: 1.01–1.43), COVID-19 severity (cigarettes: OR=1.37; 95% CI: 1.12–1.67), and harm to general health (e-cigarettes: OR=1.31; 95% CI: 1.1–1.58). Higher educational level was associated with a higher perceived risk of using cigarettes on COVID-19 infection (OR=1.2; 95% CI: 1.05–1.38) but decreased perceived risk of using cigars on COVID-19 severity (OR=0.86; 95% CI: 0.74–1.0) and general health (OR=0.81; 95% CI: 0.69–0.94). A higher household income was associated with an increased risk of severity of COVID-19 infection (cigars: OR=1.08; 95% CI: 1.01–1.17) and general health (cigars: OR=1.17; 95% CI: 1.08–1.26) for most tobacco products except cigarettes. COVID-19 deaths within the respondent’s social network were associated with the decreased perceived risk of all products to the severity of COVID-19 infection (cigarettes: OR=0.66; 95% CI: 0.52–0.85) and general health (cigarettes: OR=0.65; 95% CI: 0.5–0.85). Compared with those living in urban areas, those living in suburban areas reported decreased risk perceptions of tobacco use for COVID-19 infections (cigarettes: OR=0.72; 95% CI: 0.56–0.93).

**Table 2:** Odds of perceived risks of the use of each major tobacco/nicotine product to the COVID-19 infection (N=2097)

*Variable*		*Cigarettes OR (95% CI)*	*Cigars OR (95% CI)*	*E-cigarettes OR (95% CI)*	*Hookah OR (95% CI)*
**Gender**	Male (Ref.)	1	1	1	1
	Female	1.2 (1.01–1.43)*	1.25 (1.05–1.49)*	1.2 (1.01–1.43)*	1.05 (0.88–1.26)
**Age** (years)	18–34 (Ref.)	1	1	1	1
	35–59	0.82 (0.68–0.98)	0.91 (0.75–1.09)	0.83 (0.69–1.00)	0.87 (0.72–1.04)
	≥60	0.8 (0.51–1.24)	0.82 (0.53–1.28)	0.58 (0.37–0.93)	0.91 (0.58–1.43)
**Race/ethnicity**	White (Ref.)	1	1	1	1
	Black	1.0 (0.74–1.36)	1.09 (0.81–1.48)	1.08 (0.8–1.45)	1.06 (0.78–1.43)
	Asian	0.92 (0.66–1.27)	1.22 (0.87–1.69)	1.13 (0.81–1.57)	1.33 (0.96–1.85)
	Hispanic	1.14 (0.88–1.47)	0.97 (0.75–1.24)	0.98 (0.76–1.26)	0.93 (0.72–1.2)
	Other	1.18 (0.66–2.1)	1.19 (0.67–2.1)	0.98(0.55–1.73)	0.98 (0.56–1.74)
**Educational level**		1.2 (1.05–1.38)**	1.1 (0.96–1.26)	1.11 (0.97–1.27)	1.13 (0.99–1.30)
**Household income**		0.98 (0.91–1.05)	1 (0.93–1.07)	1.01 (0.95–1.09)	1.01 (0.94–1.08)
**COVID-19 infection for self and family**	No (Ref.)	1	1	1	1
	Yes	1.18 (0.85–1.65)	1.25 (0.90–1.73)	1.36 (0.98–1.88)	1.31 (0.95–1.82)
**COVID-19 death among social network**	No (Ref.)	1	1	1	1
	Yes	0.97 (0.77–1.22)	0.96 (0.76–1.21)	0.78 (0.62–0.99)*	0.90 (0.71–1.13)
**Urbanization level**	Urban (Ref.)	1	1	1	1
	Suburban	0.72 (0.56–0.93)**	0.72 (0.56–0.92)	0.78 (0.62–1.04)	0.72 (0.56–0.93)*
	Rural	1.16 (0.76–1.77)	0.87 (0.58–1.32)	0.9 (0.59–1.37)	0.98 (0.64–1.48)
**Use of this product in 2019**	No (Ref.)	1	1	1	1
	Yes	0.86 (0.71–1.04)	0.89 (0.72–1.10)	1.04 (0.87–1.25)	1.06 (0.83–1.34)

* p<0.05.

** p<0.01. Ref.: reference.

**Table 3:** Odds of perceived risks of the use of major tobacco/nicotine product to the severity of COVID-19 infection if infected (N=2097)

*Variable*		*Cigarettes OR (95% CI)*	*Cigars OR (95% CI)*	*E-cigarettes OR (95% CI)*	*Hookah OR (95% CI)*
**Gender**	Male (Ref.)	1	1	1	1
	Female	1.37 (1.12–1.67)**	1.39 (1.15–1.69)**	1.23 (1.03–1.48)*	1.18 (0.98–1.41)*
**Age** (years)	18–34 (Ref.)	1	1	1	1
	35–59	0.85 (0.69–1.05)	0.86 (0.71–1.05)	0.9 (0.74–1.09)	0.84 (0.69–1.01)
	≥60	0.83 (0.51–1.36)	0.72 (0.46–1.15)	0.76 (0.49–1.19)	0.80 (0.51–1.26)
**Race/ethnicity**	White (Ref.)	1	1	1	1
	Black	0.8 (0.58–1.12)	0.94 (0.68–1.3)	0.94 (0.69–1.28)	0.88 (0.65–1.2)
	Asian	1.12 (0.76–1.66)	1.33 (0.91–1.95)	1.45 (1.01–2.07)*	1.48 (1.03–2.13)*
	Hispanic	0.76 (0.58–1)*	0.84 (0.64–1.1)	0.89 (0.69–1.15)	0.87 (0.67–1.12)
	Other	1.46 (0.72–2.96)	1.09 (0.59–2.04)	1.03 (0.58–1.86)	1.01 (0.56–1.81)
**Educational level**		0.96 (0.82–1.12)	0.86 (0.74–1.0)*	0.95 (0.82–1.09)	0.93 (0.81–1.07)
**Household income**		1.07 (0.99–1.16)	1.08 (1.01–1.17)**	1.09 (1.01–1.17)*	1.09 (1.01–1.17)*
**COVID-19 infection for self and family**	No (Ref.)	1	1	1	1
	Yes	0.98 (0.68–1.39)	0.93 (0.65–1.33)	0.92 (0.65–1.31)	1.03 (0.73–1.47)
**COVID-19 death among social network**	No (Ref.)	1	1	1	1
	Yes	0.66 (0.52–0.85)**	0.67 (0.53–0.85)**	0.68 (0.53–0.85)**	0.67 (0.53–0.85)**
**Urbanization level**	Urban (Ref.)	1	1	1	1
	Suburban	0.77 (0.58–1.01)	0.82 (0.62–1.07)	0.84 (0.65–1.09)	0.77 (0.59–1.0)
	Rural	0.69 (0.44–1.08)	0.81 (0.52–1.26)	0.88 (0.57–1.34)	0.84(0.55–1.29)
**Use of this product in 2019**	No (Ref.)	1	1	1	1
	Yes	0.87 (0.70–1.07)	0.88 (0.70–1.1)	1.01 (0.83–1.21)	0.95 (0.74–1.22)

* p<0.05.

** p<0.01. Ref.: reference.

**Table 4:** Odds of perceived risks of the use of major tobacco/nicotine product to the general health (N=2097)

*Variable*		*Cigarettes OR (95% CI)*	*Cigars OR (95% CI)*	*E-cigarettes OR (95% CI)*	*Hookah OR (95% CI)*
**Gender**	Male (Ref.)	1	1	1	1
	Female	1.18 (0.95–1.47)	1.16 (0.95–1.42)	1.31 (1.1–1.58)**	0.94 (0.79–1.12)
**Age** (years)	18–34 (Ref.)	1	1	1	1
	35–59	1.28 (1.02–1.62)	1.14 (0.92–1.4)	1.13 (0.93–1.36)	1.06 (0.88–1.27)
	≥60	1.05 (0.6–1.82)	0.89 (0.55–1.45)	0.77 (0.49–1.21)	0.8 (0.51–1.24)
**Race/ethnicity**	White (Ref.)	1	1	1	1
	Black	0.72 (0.5–1.04)	0.78 (0.56–1.09)	1.04 (0.76–1.43)	0.84 (0.62–1.14)
	Asian	0.87 (0.58–1.31)	0.86 (0.59–1.24)	1.07 (0.76–1.51)	0.99 (0.71–1.38)
	Hispanic	0.51 (0.38–0.68)**	0.62 (0.48–0.82)	0.79 (0.61–1.03)	0.82 (0.64–1.06)
	Other	0.87 (0.43–1.78)	0.74 (0.4–1.37)	0.61 (0.34–1.07)	0.84 (0.48–1.49)
**Educational level**		0.87 (0.73–1.04)	0.81 (0.69–0.94)**	0.94 (0.82–1.08)	1.01 (0.88–1.15)
**Household income**		1.09 (1.0–1.19)	1.17 (1.08–1.26)**	1.11 (1.03–1.19)**	1.11 (1.03–1.19)**
**COVID-19 infection for self and family**	No (Ref.) Yes	1 1.11 (0.74–1.66)	1 1.00 (0.69–1.45)	1 1.28 (0.9–1.82)	1 1.1 (0.78–1.54)
**COVID-19 death among social network**	No (Ref.)	1	1	1	1
	Yes	0.65 (0.5–0.85)**	0.73 (0.57–0.93)*	0.74 (0.59–0.94)*	0.92 (0.73–1.16)
**Urbanization level**	Urban (Ref.)	1	1	1	1
	Suburban	0.99 (0.72–1.37)	0.95 (0.71–1.26)	0.86 (0.66–1.12)	0.99 (0.76–1.27)
	Rural	0.82 (0.5–1.34)	0.78 (0.5–1.23)	1.02 (0.66–1.58)	1.02 (0.67–1.55)
**Use of this product in 2019**	No (Ref.)	1	1	1	1
	Yes	0.93 (0.74–1.18)	0.97 (0.77–1.23)	0.76 (0.63–0.91)**	0.88 (0.69–1.12)

* p<0.05.

** p<0.01. Ref.: reference.

As Table 5 shows, for most tobacco products (particularly cigarettes and cigars), those who perceived their type(s) of tobacco-nicotine use to worsen COVID-19 severity were more likely to have decreased or quit their use (cigarettes: OR=1.65; 95% CI: 1.20–2.27; cigars: OR=1.63; 95% CI: 1.17– 2.27). No such associations were observed, however, among those perceiving their types(s) of tobacco use increases the risk of COVID-19 infection. Those who perceived risks to general health from e-cigarettes were more likely (OR=1.48; 95% CI: 1.1–1.99) to have decreased or quit their use of e-cigarettes.

**Table 5:** Odds of decreasing or quitting the use of major tobacco/nicotine products compared with increasing, initializing, or using regularly the product, with the adjustment of demographic and COVID-19 related variables

*Variable*	*Cigarettes OR (95% CI)*	*Cigars OR (95% CI)*	*E-cigarettes OR (95% CI)*	*Hookah OR (95% CI)*
Perceived risk to COVID-19 infection	1.22 (0.93–1.60)	1.18 (0.85–1.62)	1.05 (0.78–1.42)	1.05 (0.75–1.47)
Perceived risk to severity of COVID-19	1.65 (1.20–2.27)**	1.63 (1.17–2.27)**	1.20 (0.88–1.63)	1.29 (0.91–1.83)
Perceived risk to general health	1.08 (0.78–1.50)	1.05 (0.76–1.46)	1.48 (1.10–1.99)**	1.14 (0.82–1.58)

* p<0.05.

** p<0.01. Ref.: reference.

## DISCUSSION

For all three risk measures, perceived risks were higher for cigarette and cigar smoking compared with e-cigarette and hookah use, consistent with the general relative perceived risk that traditional combustible tobacco products were more harmful than non-combustible tobacco products^16-18^ and less traditional combustible products such as hookah^19,20^. For all four products, the perceived risks to general health during the pandemic were higher than the perceived risk to general health before the pandemic. Because the two risks were measured at the same time, the difference may not reflect an increase of the perceived risk of tobacco use to general health. However, such ‘perceived’ increase in perceived risks, although moderate, were consistent across the four major tobacco products and is consistent with the findings that adults generally perceive that tobacco-nicotine use is linked with increased COVID-19 incidence and severity (with COVID-19 thereby creating brand new additional harms from using those products). Unlike smoking-related cancer, heart disease, and lung disease, which generally take decades to develop, thereby obscuring their causal links, the effect of COVID-19 is much more immediate and tangible. The perceived risks of tobacco use, even from exposure to secondhand smoke or e-cigarette aerosols may increase markedly due to COVID-19.

We found that females and people with higher income or education level were more likely to endorse the risks of tobacco use. This is consistent with existing literature where perceived risk of tobacco use and many other outcomes rated higher in females than in males^21,22^. Likewise, higher income and education are associated with higher awareness of the harms of smoking and nicotine^23,24^.

It was noteworthy that reporting a COVID-19 death within a respondent’s social network was consistently associated with lower perceived risk from tobacco-nicotine use. This may be because those who had a higher perceived risk towards certain tobacco products tend to use less or do not use this product, as persons in their social network. Thus their lower tobacco use may decrease their risk of COVID-19 death. It is also possible that the salience of identifiable risk factors for COVID-19 other than tobacco use among members of one’s social networks reduces the attributions of tobacco as a causal agent for the disease.

We found that decreasing or quitting the use of certain tobacco products was associated with a greater perceived risk of severity of COVID-19 infection but not with the perceived risk of COVID-19 infection. This indicated that the concern of the severity of COVID-19 if infected was more effective as a deterrent from using the product than the concern of becoming infected. It could also be that people generally think that avoiding COVID-19 infection is largely a matter of chance or, alternatively, engaging in social distancing, mask-wearing, and handwashing, with tobacco use simply not part of the equation.

### Limitations

One limitation of this study is that the study sample was recruited from a crowdsourcing platform. Rather than a national representative sample, our participants tended to be younger and more highly educated compared to the average US adult. Secondly, as a cross-sectional study, we may not be able to accurately capture the changes in the perceived risk of tobacco use to general health. For example, as aforementioned, the perceived risks to general health before the pandemic and during the pandemic were measured at the same time, thus the difference may not reflect a real increase of the perceived risk of tobacco use to general health. Lastly, we did not capture detailed information on the history of tobacco use and current use status, which is potentially correlated with perceived risks. For example, the perceived risk is likely to be different between new and long-term users, between heavy and light users, between people who use one product, and those who are dual and poly-users.

## CONCLUSIONS

Overall, COVID-19 is increasing perceived risks among the public from using the four types of tobacco products. Consistent with the initial evidence^11-13^, the perceived risk of tobacco use on COVID-19 severity was much higher than the perceived risk of COVID-19 infection, and the majority of participants endorsed that tobacco use increases COVID-19 severity. Females and people with higher income or education level were more likely to perceive the risks of tobacco use than their counterparts. The finding that the perceived risk of tobacco use to COVID-19 infection fluctuated around 50% for various tobacco products reflects confusion among the adult public. The positive aspect is that these perceptions – whether accurate or not – appear to be prompting harm-reducing changes in tobacco product use^11,14^.

## Data Availability

The data supporting this research is available from the authors on reasonable request.
