# Supplementary material for: Perceived health risks associated with the use of tobacco and nicotine products during the COVID-19 pandemic
Source: Tob Induc Dis. 2021 Jun 9;19:46. doi: 10.18332/tid/136040 (PMC8191569; doi:10.18332/tid/136040)
Supplement: Supplementary file 1 [file TID-19-46-s1.pdf]

Supplementary

Table S1. Participants' characteristics and use status of major tobacco/nicotine products in 2019, N=2097.

Category	Item	n (%)
Gender	Female	981 (46.8)
	Male	1116 (53.2)
Age	18-34 years old	1204 (57.4)
	35-59 years old	805 (38.4)
	60 years old and above	88 (4.2)
Race/ethnicity	White	1329 (63.4)
	Black	206 (9.8)
	Asian	170 (8.1)
	Hispanic	344 (16.4)
	Others and mixed	50 (2.4)
Educational attainment	High school and below	229 (10.9)
	Above high school and below bachelor	608 (29)
	Bachelor and above	1260 (60.1)
Household income level	Less than $24,999	367 (17.5)
	$25,000 to $49,999	564 (26.9)
	$50,000 to $74,999	497 (23.7)
	$75,000 to $99,999	363 (17.3)
	$100,000 or more	306 (14.6)
[1] Urbanization level	Urban	1694 (80.8)
	Suburban	304 (14.5)
	Rural	99 (4.7)
Covid-19 infection for self and family	Yes	189 (9)
	No	1908 (91)
COVID-19 death in the social network	Yes	459 (21.9)
	No	1638 (78.1)
Cigarettes	Exclusive use	830 (39.6)
	Combined with other tobacco products	570 (27.2)
	Never use	698 (33.3)
Cigars	Exclusive use	166 (7.9)
	Combined with other tobacco products	325 (15.5)
	Never use	1606 (76.6)
E-cigarettes	Exclusive use	325 (15.5)
	Combined with other tobacco products	499 (23.8)
	Never use	1275 (60.8)
Hookah	Exclusive use	128 (6.1)
	Combined with other tobacco products	212 (10.1)
	Never use	1757 (83.8)

Notes: the urbanization level was classified using the rural-urban commuting area (RUCA) codes [15] from the home address' zip-codes, as urban (RUCA code 1). suburban (RUCA codes 2-6), and rural (RUCA codes 7-10).

Table S2. Change of using major tobacco/nicotine product during the COVID-19 pandemic, N=2097.

**Tobacco/nicotine product**	**Change of use status, n (%)**			
	Increased use level or initiliazation	Used stably	Decreased use level or quitted use	Never used
Cigarettes	556 (26.5)	296 (14.1)	495 (23.6)	749 (35.7)
Cigars	411 (19.6)	132 (6.3)	388 (18.5)	1166 (55.6)
E-cigarettes	468 (22.3)	176 (8.4)	436 (20.8)	1017 (48.5)
Hookah	361 (17.2)	92 (4.4)	384 (18.3)	1260 (60.1)

© 2021 Yang Y. et al.
